# Prevalence and clinical severity of takayasu arteritis angiographic types: a systematic review with meta-analysis

**DOI:** 10.1007/s00296-025-05983-4

**Published:** 2025-09-22

**Authors:** Nikolaos Taprantzis, Dimosthenis Chrysikos, Amir Shihada, Theodore Troupis

**Affiliations:** Department of Anatomy, Athens Medical School, National and Kapodistrian University of Athens, Mikras Asias 75, 11527 Athens, Greece

**Keywords:** Takayasu arteritis, Aorta, Prevalence, prognosis

## Abstract

Takayasu arteritis is a rare inflammatory disease that primarily affects medium- and large-sized arteries, particularly the aorta and its branches. The Hata classification defines six angiographic types based on the involved aortic segments. Clinical symptoms May vary depending on the distribution of arterial involvement. This systematic review and meta-analysis aimed to estimate the pooled prevalence of each angiographic type and evaluate their associations with clinical Manifestations.A systematic search of electronic databases was conducted to identify studies reporting angiographic classifications and clinical symptoms in patients with Takayasu arteritis. Pooled prevalence estimates were calculated using R software, including subgroup analyses by geographic area and imaging modality. Meta-regression was used to assess associations between angiographic types and specific clinical features.Type V was the most common angiographic subtype, with a pooled prevalence of 43.49%, while type III was the least common, 5.32%. Subgroup analyses showed statistically significant differences only for type IIb, based on modality types. Meta-regression revealed significant correlations between angiographic types and clinical symptoms, with Type V exhibiting the greatest severity, and types IIb and III the lowest.This meta-analysis highlights the varying distribution of angiographic types of Takayasu arteritis and their significant associations with clinical symptoms, which may guide prognostic and management strategies.

**Supplementary Information:**

The online version contains supplementary material available at 10.1007/s00296-025-05983-4.

## Introduction

Takayasu arteritis, or else known as the pulseless disease, refers to an inflammatory-based medical condition, which is characterized by the presence of damage to the medium-sized and large-sized arteries, as well as to their respective branches [1]. In general, this inflammatory disease is considered an uncommon medical entity, with an approximate incidence rate of 1 or 2 per one million, while the majority of affected individuals are female [2]. The prevalence superiority of female against male patients has been supported by data from many countries across different continents [2]. In most cases, Takayasu arteritis begins its clinical profile during the 2nd or 3rd decade of the patient’s life, solidifying itself as a disease of young individuals [3].

Diagnostic evaluation of Takayasu Arteritis is performed though a variety of imaging techniques like Conventional Angiography, Positron Emission Tomography (PET), Computed Tomography (CT), and Magnetic Resonance Imaging (MRI), with each one of them presenting with different advantages and disadvantages regarding their accessibility, ease of use, and depictive limitations [4]. Digital Subtraction Angiography (DSA) is considered the gold standard for diagnosis, while CTA and MRI are associated with better results regarding the monitoring and surveillance of the patient’s condition [5]. Among the Main symptoms of this inflammatory disease, absent or diminished pulses, as well as vascular bruits are reported in over 80% of the affected patients, while hypertension, ocular problems, neurological features and others, can also be observed less often [6, 7] After the initial attempt to classify the disease in 1977, Hata et al. improved the system in 1996 creating six categories (I-V), that reflected the affected segments of the aorta [8].

Even though there are systematic reviews, with/or without meta-analysis, that investigate the impact of Takayasu arteritis on the presence and development of specific clinical manifestations, such as stroke or hypertension, an analysis that assesses a larger group of patient symptoms, in order to determine the clinical severity associated with each angiographic type, does not exist. Additionally, we could not identify any meta-analyses that calculated the pooled prevalence percentages of each angiographic type, as well as their variability across different diagnostic modalities and geographical areas. Thus, this systematic review with meta-analysis aims to explore this gap and provide useful information regarding the severity and clinical profile of Takayasu arteritis.

## Materials and methods

### Study selection

We systematically searched through the PubMed, Embase, Web of Science, Directory of Open Access Journals, and Scopus databases using key words like “Takayasu arteritis”, “angiographic type”, “prevalence of Takayasu arteritis”, “angiographies”, “clinical symptoms, signs and manifestations”, “imaging study”, “radiologic features”, “clinical characteristics”, “Takayasu arteritis classification frequency”, order to collect data that would fit the criteria of our review. This database search took place from May 2025 to August 2025. Since this paper used only published data, any Institutional review Board approval or any other consent were not needed. Studies published between January 1 st 1996 and August 1 st 2025, were included in this study.

Two reviewers (DC and NT) independently screened the titles and abstracts of all records retrieved through the database searches. Studies that appeared relevant were then reviewed in full text. Disagreements at either stage were resolved through discussion and consensus between the two reviewers. No automation tools or machine learning algorithms were used in the selection process. Duplicates were removed manually using Microsoft Excel. This systematic review with meta-analysis adhered to Gasparyan et al. recommendation on comprehensive search strategies [9].

### Inclusion criteria

The inclusion of studies, that were analyzed in this review, was based on a fixed collection of inclusion and exclusion criteria. Studies were included if (1) participants had been diagnosed with Takayasu arteritis, (2) participants were assessed and grouped according to their angiographic type of Takayasu arteritis, based on Hata’s classification.

These inclusion criteria were established to provide a consistent and reliable reference framework, minimizing confusion and reporting discrepancies across studies. The Hata classification system was chosen because it is the most widely used and recognized method for categorizing Takayasu arteritis. Its broad acceptance ensures greater consistency in reported data and maximizes the number of studies eligible for inclusion.

### Exclusion criteria

Studies were excluded if (1) participants were grouped according to a different classification system, (2) an angiographic classification system was not used at all, (3) animal studies or conference abstracts.

### Data extraction

Two reviewers (DC and NT) independently extracted data from the included studies using a standardized data extraction form in Microsoft Excel. The extracted data included raw count data for types of arterial involvement and clinical symptoms. After completing data extraction independently, the reviewers compared their datasets and resolved discrepancies through consensus. No automated tools were used in the data extraction process.

### Data sought

The primary outcomes of interest were the anatomical patterns of arterial involvement in Takayasu arteritis, defined by the Hata classification types (Types I, IIa, IIb, III, IV, and V). Secondary outcomes included clinical symptoms associated with the disease, such as upper/lower limb claudication, hypertension, visual disturbances, headache, chest pain, fatigue, syncope, stroke, bruit, palpitations, pulselessness, and arthralgia.

After an initial search of the literature, regarding the most common clinical manifestations of Takayasu arteritis, it was decided that this group of patient symptoms, that represented different categories of disease features (such as ischemic, cardiovascular, systemic) would be included [3, 10]. The goal was to explore potential associations between angiographic involvement and the broad clinical spectrum of the disease. This approach was exploratory, without a strict hypothesis regarding which angiographic types would correlate with specific symptoms, as existing literature on such correlations remains limited.

For each included study, we aimed to extract all available results related to the predefined outcome domains. If multiple time points or subgroups were reported, we prioritized baseline data. Where outcomes were reported using different terms or formats, data were standardized based on consistent definitions across studies.

The two most well-known classification systems for the different angiographic types of Takayasu arteritis belong to Hata et al. and Nasu, with the former dividing this clinical entity into 6 different categories, while the latter into four.

As far as Hata et al.’s classification is concerned:

Type I: Includes the branches of the aortic arch.

Type IIa: Includes the ascending aorta, the aortic arch and its branches.

Type IIb: Includes the ascending aorta, the aortic arch and its branches, the thoracic, and descending aorta.

Type III: Includes the thoracic, descending, abdominal aorta and/or renal arteries.

Type IV: Includes the abdominal aorta and/or renal arteries.

Type V: Combination of IIb and IV.

 [8].

### Other variables extracted

In addition to the primary and secondary outcomes, we extracted key study characteristics, including first author, year of publication, and total number of participants. Furthermore, study characteristics, such as modality group and region of data collection, were extracted so as to investigate any differences in pooled prevalence between these factors. The geographical settings included Europe, America, Asia, and Oceania. On the other hand, the modality group refers to the type of investigation that that performed so as to find and report the different angiographic type. Precisely, studies were divided into cross-sectional Imaging-based examination (e.g. CT, MRA, MDCT), Conventional angiography-based, and mixed approach.

### Risk of bias assessment

Risk of bias for each included study was assessed using the Hoy et al. tool for prevalence studies [11]. Two reviewers (DC and NT) conducted the assessments independently. Each domain of the tool was rated separately, and an overall judgment of risk (low, moderate, or high) was assigned per study. In cases of disagreement, a consensus was reached through discussion.

### Statistical methods

We conducted a meta-analysis using R (version 4.3.2) and RStudio, utilizing the “meta”, “metafor”, “metaprop”, and “dmetar” packages for statistical processing. Pooled prevalence estimates for each angiographic type of Takayasu arteritis were calculated using the inverse variance method with a logit (PLOGIT) transformation to stabilize variances, particularly for proportions close to 0 or 1. A random-effects model was applied using the DerSimonian–Laird estimator (DL) for between-study variance (τ²) and the Hartung–Knapp adjustment (HK) to produce more robust confidence intervals. Subgroup analyses were performed to investigate differences in prevalence by imaging modality and geographic region. Univariate meta-regression was conducted using a random-effects model with Restricted Maximum Likelihood (REML) estimation to evaluate associations between the prevalence of each angiographic type and clinical manifestations. In this analysis, the effect measure was the regression coefficient (β) indicating the change in prevalence per unit change in moderator variables, along with corresponding p-values and 95% confidence intervals. Moderator effects were tested using z-statistics, and model fit was summarized with QM and QE statistics. Heterogeneity was assessed using Cochran’s Q test and the Higgins I² statistic, with I² values interpreted as follows: 0–40% (low), 30–60% (moderate), 50–90% (substantial), and 75–100% (considerable heterogeneity. Statistical significance was set at *p* < 0.05 for all analyses.

Results from individual studies, including prevalence estimates for each angiographic type and estimates for clinical Manifestation, were tabulated in summary tables in order to display the pooled prevalence estimates, 95% confidence intervals for each angiographic type and subgroup (by imaging modality and geographic region), as well as the univariate analyses estimate. P-values were also included in these tables. All tables were constructed to clearly present study-level data and synthesized outcomes.

To evaluate potential reporting biases affecting the synthesis, we assessed publication bias using the Luis Furuya–Kanamori (LFK) index [12]. An absolute LFK index greater than 1.0 was considered indicative of minor asymmetry, while values exceeding 2.0 suggested major asymmetry and potential publication bias. This method allowed for the detection of small-study effects and other forms of reporting bias that might influence the meta-analytic estimates. Additionally, to assess the presence of publication bias, Peter’s test, along with the corresponding plots, were performed for each angiographic type of Takayasu arteritis [13]. Finally, a leave-one-out sensitivity analysis was also conducted in order to investigate any potential differences in pooled prevalence of each type.

To account for the increased risk of false-positive findings due to multiple hypothesis testing, we applied the Benjamini–Hochberg procedure to control the false discovery rate (FDR) [14]. This correction was applied to the p-values obtained from the univariate analyses investigating the association between angiographic types and clinical symptoms.

### Assessment of certainty in the body of evidence

The overall certainty (or confidence) in the evidence for each outcome was qualitatively evaluated by considering the risk of bias of included studies, consistency of results, outcomes from Peter’s test, the sensitivity analysis, the corrected p-values from the Benjamini-Hochberg procedure, and the LFK index.

## Results

### Study characteristics

**Fig. 1:**
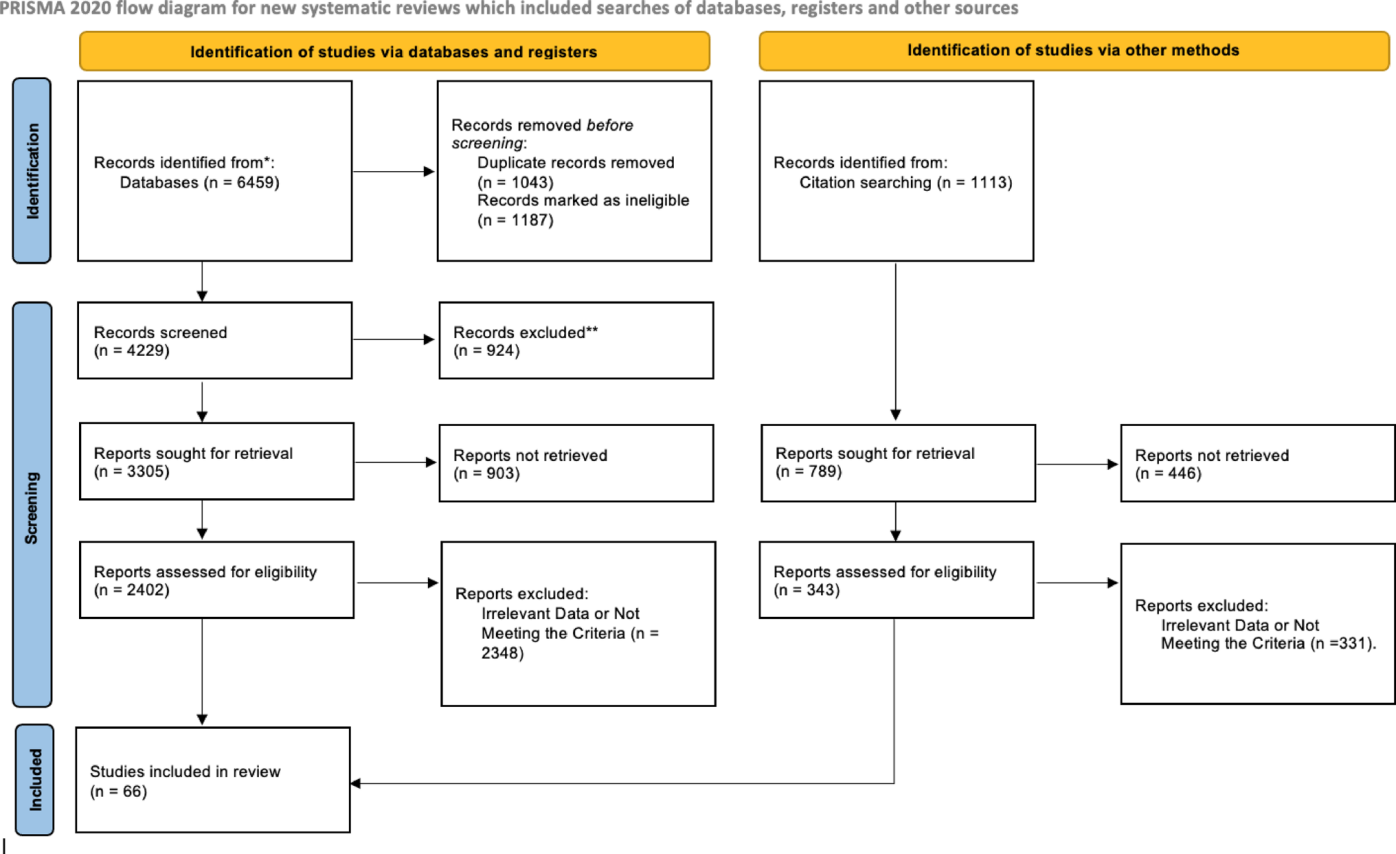
PRISMA flow diagram [15]

After the completion of our systematic search, 7572 studies were identified from databases and citation searching. Following the removal of duplicates and articles that did not meet the predefined criteria, set by us, 2745 studies were assessed for eligibility. Finally, it was decided that 66 studies would be included in the systematic review [Figure 1].

Among the included studies, 189.45 patients was the mean sample per study. Furthermore, 9 studies were characterized as Conventional Angiography-based, 19 studies utilized the cross-sectional imaging method, 33 studies used both, and were considered mixed, while in 5 of the them the diagnostic method was not clearly stated. 10 studies belonged to the American population, 9 to the European, only 1 to Oceanic, and 44 to the Asian. The study characteristics are presented in Table 1.

**Table 1:** Study characteristics

Study	Year	Modality	Area	Patients	Risk of bias
Gudbrandsson [16]	2017	Mixed	Europe	77	Low risk
Gloor [17]	2021	Imaging	Europe	31	Moderate
Saritas [18]	2016	N/A	Asia	23	Moderate
Dreyer [19]	2010	N/A	Europe	19	Low risk
Soto [20]	2007	Mixed	America	110	High risk
Karageorgaki [21]	2008	Mixed	Europe	42	Low risk
Bicakcigil [22]	2009	Angiography	Asia	248	Low risk
Schmidt [23]	2013	Mixed	America	126	Moderate
Lim [24]	2015	Mixed	Asia	294	Moderate
Sato [25]	1998	Angiography	America	73	High risk
Zhou [26]	2023	Mixed	Asia	852	Low risk
Sheikzadeh [27]	2002	Angiography	Asia	78	Moderate
Canas [28]	1998	N/A	America	35	High risk
Setty [29]	2016	Angiography	America	50	Moderate
Sun [30]	2017	Mixed	Asia	411	Moderate
Lee [31]	2012	Mixed	Asia	204	Low risk
Cong [32]	2010	Angiography	Asia	125	High risk
Park [33]	2005	Angiography	Asia	108	Moderate
Suwanwela [34]	1998	Mixed	Asia	63	Moderate
Alvarez [35]	2019	Mixed	America	40	Low risk
Ong [36]	2024	Mixed	Asia	85	Low risk
Watanabe [37]	2015	N/A	Asia	1335	Low risk
Esen [38]	2019	Imaging	Asia	17	Low risk
Markin [39]	2017	Mixed	Oceania	17	Moderate
Comarmond [40]	2017	Imaging	Europe	257	Moderate
Danda [41]	2020	Angiography	Asia	585	Moderate
Kong [42]	2025	Imaging	Asia	239	Low risk
Karabacak [43]	2021	Mixed	Asia	141	Low risk
Oliveira [44]	2022	Mixed	America	66	High risk
Mahdavi [45]	2020	Mixed	Asia	75	Moderate
Petrovic [46]	2008	Mixed	Europe	16	High risk
Arnaud [47]	2010	Mixed	Europe	79	Low risk
Tamartash [48]	2020	Imaging	Asia	143	Moderate
Li [49]	2019	Mixed	America	225	High risk
Goel [50]	2019	Mixed	Asia	581	Moderate
Kim [51]	2005	Imaging	Asia	27	Low risk
De Paula [52]	2013	N/A	America	18	Moderate
Eleftheriou [53]	2015	Mixed	Europe	11	Moderate
Li J [54]	2017	Mixed	Asia	411	High risk
Cheng [55]	2019	Mixed	Asia	397	Low risk
Figueiroa [56]	2023	Imaging	America	30	Moderate
Zhang [57]	2018	Mixed	Asia	1069	Low risk
Wong [58]	2018	Mixed	Asia	75	Low risk
Zhang Y [59]	2019	Imaging	Asia	533	Moderate
Khan [60]	2022	Imaging	Asia	18	Low risk
Clemente [61]	2016	Mixed	America	71	Moderate
Johnson [62]	2021	Mixed	America	12	Moderate
Lei [63]	2019	Mixed	Asia	38	Moderate
Kelesoglu [64]	2021	Mixed	Asia	97	Moderate
Hegde [65]	2020	Imaging	Asia	30	High risk
Xi [66]	2021	Imaging	Asia	126	Low risk
Misra [67]	2024	Mixed	Asia	191	Low risk
Zhang [68]	2023	Mixed	Asia	411	Low risk
Misra [69]	2022	Imaging	Asia	191	Moderate
Mukoyoma [70]	2021	Imaging	Asia	166	Moderate
Zhang [71]	2024	Mixed	Asia	153	High risk
He [72]	2019	Mixed	Asia	194	High risk
Ren [73]	2020	Imaging	Asia	103	Moderate
Li [74]	2016	N/A	Asia	50	Low risk
Kwon [75]	2018	Imaging	Asia	268	Low risk
Ma [76]	2023	Imaging	Asia	82	Low risk
Kalfa [77]	2023	Angiography	Asia	70	Moderate
Fan [78]	2019	Mixed	Asia	101	Moderate
Chen [79]	2018	Mixed	Asia	411	Low risk
Wang [80]	2019	Mixed	Asia	130	High risk
Kong [81]	2020	Imaging	Asia	216	High risk

### Type I prevalence

The pooled prevalence of the Type I Hata Classifications was calculated at 23.50% with 95% CI: 0.2111;0.2608. The LFK index for Type I was 0.441.

#### Type I subgroup analyses

Moreover, subgroup analysis based on Modality group depicted that the pooled prevalence with Angiography was 26.64% with 95% CI: 0.1782;0.3781, 23.09% with Imaging methods and 95%CI: 0.1916;0.2755, while Mixed Modality had 21.48% and 95% CI: 0.1865;0.2459.

Based on geographical area, pooled prevalence was 27.51% in Europe with 95% CI: 0.2004;0.3649, 23.62% in Asia with 95% CI: 0.2105;0.2641, 20.48% in America with 95% CI: 0.1340;0.3000, while one study was conducted in Oceania with prevalence of 23.53%. However, neither of these moderators were statistically significant.

### Type I clinical association

The meta-analysis of associations between Type I and clinical manifestations showed both positive and negative effect estimates. Positive estimates included upper limb claudication (0.2841; 95% CI: 0.0531 to 0.5151; *p* = 0.1033), down limb claudication (0.4350; 95% CI: −0.0483 to 0.9183; *p* = 0.2525), fatigue (0.0202; 95% CI: −0.2165 to 0.2570; *p* = 0.9391), headache (0.1865; 95% CI: −0.0352 to 0.4082; *p* = 0.2579), pulselessness (0.1218; 95% CI: −0.0762 to 0.3197; *p* = 0.4937), visual problems (0.1658; 95% CI: −0.2120 to 0.5437; *p* = 0.5627), stroke (0.3102; 95% CI: −0.2845 to 0.9049; *p* = 0.4982), and arthralgia (0.0490; 95% CI: −0.2140 to 0.3120; *p* = 0.8450).

Negative estimates included palpitation (−0.2093; 95% CI: −0.5816 to 0.1630; *p* = 0.4982), chest pain (−0.2994; 95% CI: −0.5827 to −0.0162; *p* = 0.1659), hypertension (−0.1711; 95% CI: −0.3061 to −0.0361; *p* = 0.1033), vascular bruits (−0.0051; 95% CI: −0.1769 to 0.1667; *p* = 0.9535), and syncope (−0.1267; 95% CI: −0.4529 to 0.1996; *p* = 0.5877).

### Type IIA prevalence

The pooled prevalence of the Type IIa Hata Classifications was calculated at 6.61% with 95% CI: 0.0562;0.0775. The LFK index for Type IIa was − 1.358.

#### Type IIA subgroup analyses

Moreover, subgroup analysis based on Modality group depicted that the pooled prevalence with Angiography was 3.42% with 95% CI: 0.0154;0.0744, 7.15% with Imaging methods and 95%CI: 0.0545;0.0.0932, while Mixed Modality had 6.47% and 95% CI: 0.0534;0.0783.

Based on geographical area, pooled prevalence was 9.23% in Europe with 95% CI: 0.0702;0.1204, 6.05% in Asia with 95% CI: 0.0497;0.0734, 7.65% in America with 95% CI: 0.0478;0.1201, while one study was conducted in Oceania with prevalence of 5.88%. However, neither of these moderators were statistically significant.

### Type IIA clinical association

The meta-analysis of associations between Type IIa and clinical manifestations showed a mix of positive and negative estimates. Positive estimates, though all non-significant, included upper limb claudication (0.0459; 95% CI: −0.0952 to 0.1871; *p* = 0.6805), down limb claudication (0.0101; 95% CI: −0.2547 to 0.2749; *p* = 0.9403), chest pain (0.0623; 95% CI: −0.0418 to 0.1665; *p* = 0.6258), stroke (0.2249; 95% CI: −0.0712 to 0.5210; *p* = 0.5915), and vascular bruit (0.0215; 95% CI: −0.0421 to 0.0850; *p* = 0.6805).

Negative estimates included fatigue (−0.0163; 95% CI: −0.1372 to 0.1046; *p* = 0.8578), palpitation (−0.1356; 95% CI: −0.3114 to 0.0403; *p* = 0.5915), headache (−0.0382; 95% CI: −0.1164 to 0.0400; *p* = 0.6805), pulselessness (−0.0373; 95% CI: −0.1229 to 0.0483; *p* = 0.6805), visual problems (−0.1081; 95% CI: −0.2698 to 0.0537; *p* = 0.6184), hypertension (−0.0799; 95% CI: −0.1365 to −0.0233; *p* = 0.0741), syncope (−0.0443; 95% CI: −0.2168 to 0.1281; *p* = 0.7261), and arthralgia (−0.0206; 95% CI: −0.0823 to 0.0411; *p* = 0.6805).

### Type IIB prevalence

The pooled prevalence of the Type IIb Hata Classifications was calculated at 8.11% with 95% CI: 0.0680;0.0965. The LFK index for Type IIb was − 0.432.

#### Type IIB subgroup analyses

Moreover, subgroup analysis based on Modality group depicted that the pooled prevalence with Angiography was 3.65% with 95% CI: 0.0273; 0.0487, 11.66% with Imaging methods and 95%CI: 0.0873; 0.1541, while Mixed Modality had 7.51% and 95% CI: 0.0605; 0.0928.

Based on geographical area, pooled prevalence was 11.22% in Europe with 95% CI: 0.0746; 0.1654, 7.86% in Asia with 95% CI: 0.0639; 0.0963, 7.03% in America with 95% CI: 0.0434; 0.1119, while one study was conducted in Oceania with prevalence of 23.53%. Only the “Modality” moderator were statistically significant, with p-value < 0.0001.

### Type IIB clinical association

The meta-analysis of associations between Type IIb and clinical manifestations showed mostly negative estimates, including significant associations for pulselessness (−0.1184; 95% CI: −0.1935 to −0.0434; *p* = 0.0130) and visual problems (−0.2471; 95% CI: −0.4006 to −0.0936; *p* = 0.0130). Other negative estimates were noted for upper limb claudication (−0.0439; 95% CI: −0.1871 to 0.0992; *p* = 0.7113), down limb claudication (−0.2098; 95% CI: −0.4005 to −0.0192; *p* = 0.1007), fatigue (−0.1156; 95% CI: −0.2656 to 0.0344; *p* = 0.2836), palpitation (−0.1358; 95% CI: −0.4253 to 0.1537; *p* = 0.5814), headache (−0.1145; 95% CI: −0.2137 to −0.0153; *p* = 0.1007), hypertension (−0.0658; 95% CI: −0.1460 to 0.0144; *p* = 0.2802), stroke (−0.0527; 95% CI: −0.3279 to 0.2224; *p* = 0.7662), vascular bruit (−0.0460; 95% CI: −0.1391 to 0.0471; *p* = 0.3326), syncope (−0.0717; 95% CI: −0.2575 to 0.1141; *p* = 0.6994), and arthralgia (−0.0001; 95% CI: −0.0901 to 0.0899; *p* = 0.9989).

The only positive, though non-significant, estimate was observed for chest pain (0.0457; 95% CI: −0.1305 to 0.2220; *p* = 0.7220).

### Type III prevalence

The pooled prevalence of the Type III Hata Classifications was calculated at 5.32% with 95% CI: 0.0442; 0.0638. The LFK index for type III was − 0.202.

#### Type III subgroup analyses

Moreover, subgroup analysis based on Modality group depicted that the pooled prevalence with Angiography was 6.02% with 95% CI: 0.0263; 0.1319, 5.26% with Imaging methods and 95%CI: 0.0396; 0.0696, while Mixed Modality had 4.86% and 95% CI: 0.0399; 0.0591.

Based on geographical area, pooled prevalence was 4.98% in Europe with 95% CI: 0.0162; 0.1431, 5.06% in Asia with 95% CI: 0.0435; 0.0602, 6.81% in America with 95% CI: 0.0398; 0.1141, while one study was conducted in Oceania with prevalence of 2.78%. Both of these moderators were not statistically significant.

### Type III clinical association

The meta-analysis of associations between Type III Takayasu arteritis and clinical manifestations revealed mostly negative estimates, including significant associations for upper limb claudication (−0.0622; 95% CI: −0.0994 to −0.0251; *p* = 0.0130) and pulselessness (−0.0547; 95% CI: −0.0947 to −0.0146; *p* = 0.0487). Other negative but non-significant estimates included down limb claudication (−0.0615; 95% CI: −0.1407 to 0.0177; *p* = 0.2083), fatigue (−0.0895; 95% CI: −0.1722 to −0.0068; *p* = 0.1098), palpitation (−0.1806; 95% CI: −0.3724 to 0.0111; *p* = 0.1404), headache (−0.0676; 95% CI: −0.1294 to −0.0058; *p* = 0.1098), visual problems (−0.0838; 95% CI: −0.2219 to 0.0543; *p* = 0.3048), stroke (−0.0973; 95% CI: −0.2917 to 0.0971; *p* = 0.3859), syncope (−0.0619; 95% CI: −0.2066 to 0.0827; *p* = 0.4345), and arthralgia (−0.0043; 95% CI: −0.0093 to 0.0006; *p* = 0.1565).

Positive estimates, none reaching significance, were observed for chest pain (0.1211; 95% CI: −0.0061 to 0.2483; *p* = 0.1404), hypertension (0.0313; 95% CI: −0.0137 to 0.0763; *p* = 0.2496), and vascular bruit (0.0018; 95% CI: −0.0539 to 0.0576; *p* = 0.9490).

### Type IV prevalence

The pooled prevalence of the Type IV Hata Classifications was calculated at 8.25% with 95% CI: 0.0677; 0.1002. The LFK index for type IV was − 0.853.

#### Type IV subgroup analyses

Moreover, subgroup analysis based on Modality group depicted that the pooled prevalence with Angiography was 11.11% with 95% CI: 0.0681; 0.1763, 5.74% with Imaging methods and 95%CI: 0.0367; 0.0887, while Mixed Modality had 9.08% and 95% CI: 0.0705; 0.1162.

Based on geographical area, pooled prevalence was 6.42% in Europe with 95% CI: 0.0454; 0.0901, 8.54% in Asia with 95% CI: 0.0678; 0.1070, 7.69% in America with 95% CI: 0.0428; 0.1345, while one study was conducted in Oceania with prevalence of 5.88%. Both of these moderators were not statistically significant.

### Type IV clinical association

The meta-analysis of associations between Type IV Takayasu arteritis and clinical manifestations showed both negative and positive estimates. Positive estimates were observed for chest pain (0.1061; 95% CI: −0.1264 to 0.3387; *p* = 0.5360), pulselessness (0.0370; 95% CI: −0.1186 to 0.1926; *p* = 0.8335), visual problems (0.0057; 95% CI: −0.2447 to 0.2561; *p* = 0.9909), and hypertension (0.1517; 95% CI: 0.0541 to 0.2494; *p* = 0.0149).

Negative associations included upper limb claudication (−0.0961; 95% CI: −0.1358 to −0.0563; *p* = 0.0117), down limb claudication (−0.0941; 95% CI: −0.1833 to −0.0049; *p* = 0.1672), fatigue (−0.0921; 95% CI: −0.2476 to 0.0635; *p* = 0.5231), palpitation (−0.2711; 95% CI: −0.5474 to 0.0053; *p* = 0.1771), headache (−0.0623; 95% CI: −0.1747 to 0.0501; *p* = 0.5231), stroke (−0.1675; 95% CI: −0.4724 to 0.1375; *p* = 0.5231), vascular bruit (−0.0008; 95% CI: −0.1314 to 0.1299; *p* = 0.9909), syncope (−0.0188; 95% CI: −0.2673 to 0.2298; *p* = 0.8823), and arthralgia (−0.0878; 95% CI: −0.2650 to 0.0894; *p* = 0.5360).

### Type V prevalence

The pooled prevalence of the Type V Hata Classifications was calculated at 43.49% with 95% CI: 0.4002; 0.4703. The LFK index for type V was 1.271.

#### Type V subgroup analyses

Moreover, subgroup analysis based on Modality group depicted that the pooled prevalence with Angiography was 35.99% with 95% CI: 0.2740; 0.4558, 45.30% with Imaging methods and 95%CI: 0.4129; 0.4938, while Mixed Modality had 45.98% and 95% CI: 0.4045; 0.5161.

Based on geographical area, pooled prevalence was 44.50% in Europe with 95% CI: 0.3281; 0.5683, 45.88% in Asia with 95% CI: 0.4256; 0.4925, 31.54% in America with 95% CI: 0.2081; 0.4469, while one study was conducted in Oceania with prevalence of 41,18%. Neither of the two moderators were statistically significant.

### Type V clinical association

The meta-analysis of associations between Type V Takayasu arteritis and clinical manifestations revealed both positive and negative trends. Positive estimates were observed for palpitation (0.9739; 95% CI: 0.5197 to 1.4281; *p* = 0.0013), visual problems (0.3931; 95% CI: −0.1029 to 0.8890; *p* = 0.2632), hypertension (0.1522; 95% CI: −0.0368 to 0.3414; *p* = 0.2632), syncope (0.2035; 95% CI: −0.2572 to 0.6642; *p* = 0.5584), arthralgia (0.1822; 95% CI: −0.2184 to 0.5828; *p* = 0.5584), headache (0.0422; 95% CI: −0.2408 to 0.3251; *p* = 0.8776), and fatigue (0.0137; 95% CI: −0.3661 to 0.3934; *p* = 0.9438).

Negative estimates were found for stroke (−0.7685; 95% CI: −1.3550 to −0.1820; *p* = 0.0663), chest pain (−0.4087; 95% CI: −0.8293 to 0.0118; *p* = 0.2461), upper limb claudication (−0.2871; 95% CI: −0.6505 to 0.0763; *p* = 0.2632), lower limb claudication (−0.2396; 95% CI: −0.9054 to 0.4261; *p* = 0.6246), pulselessness (−0.1060; 95% CI: −0.3193 to 0.1074; *p* = 0.5584), and vascular bruit (−0.0270; 95% CI: −0.2468 to 0.1929; *p* = 0.8776) (Tables 2 and 3).

**Table 2:** Statistical results of the subgroup analysis

Parameters	Type I (%)	Type IIa (%)	Type IIb (%)	Type III (%)	Type IV (%)	Type V (%)
Europe	27.51	9.23	11.22	4.98	6.42	44.50
America	20.48	7.65	7.03	6.81	7.69	31.54
Asia	23.62	6.05	7.86	5.06	8.54	45.88
Oceania	23.53	5.88	23.53	2.78	5.88	41.18
p-value	0.7025	0.0983	0.0609	0.7321	0.5844	0.2225
Conventional angiography	26.64	3.42	3.65	6.02	11.11	35.99
Imaging	23.09	7.15	11.66	5.26	5.74	45.30
Mixed	21.48	6.47	7.51	4.86	9.08	45.98
p-value	0.5420	0.2142	< 0.001	0.8169	0.1024	0.1765

**Table 3:** Statistical results from the meta regressions

Parameters	Type I	Type IIa	Type IIb	Type III	Type IV	Type V
Upper limb claudication	0.2841	0.0459	−0.0439	−0.0622	−0.0961	−0.2871
p-value	0.0159*	0.5235	0.5472	0.0010*	< 0.001*	0.1215
Corrected p-value	0.1033	0.6805	0.7113	0.0130*	0.0117*	0.2632
Down limb claudication	0.4350	0.0101	−0.2098	−0.0615	−0.0941	−0.2396
p-value	0.0777	0.9403	0.0310*	0.1282	0.0386*	0.4805
Corrected p-value	0.2525	0.9403	0.1007	0.2083	0.1672	0.6246
Fatigue	0.0202	−0.0163	−0.1156	−0.0895	−0.0921	0.0137
p-value	0.8669	0.7919	0.1309	0.0338*	0.2460	0.9438
Corrected p-value	0.9391	0.8578	0.2836	0.1098	0.5231	0.9438
Palpitation	−0.2093	−0.1356	−0.1358	−0.1806	−0.2711	0.9739
p-value	0.2706	0.1308	0.3578	0.0648	0.0545	< 0.0001*
Corrected p-value	0.4982	0.5915	0.5814	0.1404	0.1771	0.0013*
Headache	0.1865	−0.0382	−0.1145	−0.0676	−0.0623	0.0422
p-value	0.0992	0.3384	0.0236*	0.0321*	0.2775	0.7703
Corrected p-value	0.2579	0.6805	0.1007	0.1098	0.5231	0.8776
Chest Pain	−0.2994	0.0623	0.0457	0.1211	0.1061	−0.4087
p-value	0.0383*	0.2407	0.6110	0.0620	0.3711	0.0568
Corrected p-value	0.1659	0.6258	0.7220	0.1404	0.5360	0.2461
Pulselessness	0.1218	−0.0373	−0.1184	−0.0547	0.0370	−0.1060
p-value	0.2279	0.3929	0.0020*	0.0075*	0.6412	0.3303
Corrected p-value	0.4937	0.6805	0.0130*	0.0487*	0.8335	0.5584
Visual Problems	0.1658	−0.1081	−0.2471	−0.0838	0.0057	0.3931
p-value	0.3896	0.1903	0.0016*	0.2345	0.9644	0.1203
Corrected p-value	0.5627	0.6184	0.0130*	0.3048	0.9909	0.2632
Hypertension	−0.1711	−0.0799	−0.0658	0.0313	0.1517	0.1522
p-value	0.0130*	0.0057*	0.1078	0.1728	0.0023*	0.1145
Corrected p-value	0.1033	0.0741	0.2802	0.2496	0.0149*	0.2632
Stroke	0.3102	0.2249	−0.0527	−0.0973	−0.1675	−0.7685
p-value	0.3066	0.1365	0.7073	0.3266	0.2817	0.0102*
Corrected p-value	0.4982	0.5915	0.7662	0.3859	0.5231	0.0663
Vascular Bruit	−0.0051	0.0215	−0.0460	0.0018	−0.0008	−0.0270
p-value	0.9535	0.5084	0.3326	0.9490	0.9909	0.8101
Corrected p-value	0.9535	0.6805	0.5814	0.9490	0.9909	0.8776
Syncope	−0.1267	−0.0443	−0.0717	−0.0619	−0.0188	0.2035
p-value	0.4467	0.6144	0.4496	0.4011	0.8823	0.3866
Corrected p-value	0.5877	0.7261	0.6994	0.4345	0.9909	0.5584
Arthralgia	0.0490	−0.0206	−0.0001	−0.0043	−0.0878	0.1822
p-value	0.7150	0.5120	0.9989	0.0843	0.3312	0.3727
Corrected p-value	0.8450	0.6805	0.9989	0.1565	0.5360	0.5584

The geographic and modality-based distribution of Takayasu arteritis types shows distinct patterns. Type I was most prevalent in Europe (27.51%) and most frequently identified through conventional angiography (26.64%). Type IIa and Type IIb were also most common in Europe, with prevalence rates of 9.23% and 11.22%, respectively, and were most often reported using mixed imaging modalities (7.15% and 11.66%). Type III was most prevalent in America (6.81%) and most frequently detected by conventional angiography (6.02%). Type IV showed the highest prevalence in Asia (8.54%), with conventional angiography being the leading modality (11.11%). Type V had the highest overall prevalence, particularly in Asia (45.88%), and was most commonly identified using Mixed diagnostic techniques (45.98%).

### Sensitivity analysis

A leave-one-out sensitivity analysis showed that no individual study unduly influenced the pooled prevalence estimates for any type. For Type I, the estimate ranged between 23.44% and 23.94%, with high heterogeneity (I² ≈ 82%). For Type IIa, estimates ranged from 6.37 to 6.76% (I² ≈ 82%), and for Type IIb, from 7.92 to 8.20% (I² ≈ 83%). For Type III, the estimate ranged between 5.13% and 5.40% (I² ≈ 78%), and for Type IV, between 8.26% and 8.51% (I² ≈ 87%). Finally, for Type V, the estimate ranged from 43.24 to 44.15%, with the highest heterogeneity (I² ≈ 92%).

### Risk of bias assessment

Each study that was included in this analysis, was assessed according to the Hoy et al. Risk of bias tool. The detailed bias assessment of the methodological quality of the studies is presented in Supplementary File 1 [Supplementary File 1]. Additionally, Peter’s test for funnel plot asymmetry did not show statistically significant evidence for any angiographic type of Takayasu arteritis (all p-values > 0.05), indicating no substantial publication bias in the pooled prevalence estimates [Supplementary File 2].

## Discussion

This systematic review with meta-analysis calculated and presented the pooled prevalence of each angiographic type of Takayasu Arteritis, according to the Hata classification, across different geographical areas, as well as separate diagnostic techniques. Additionally, analysis regarding the association between each angiographic type and a variety of different clinical manifestations was conducted. While no other systematic review with meta-analysis has investigated the overall pooled prevalence of each angiographic type, along with their clinical severity, some other studies have reported their conclusion regarding the severity and clinical associations, in different patient settings.

The results from this study showed that the most common angiographic type was Type V with a pooled prevalence of 43.49%, while the rarest was Type III with 5.32%. The subgroup analysis showed statistically significant results.

only in Type IIb the modality groups.

As far as the univariate meta regression and the p-value correction is concerned, a statistically significant correlation was depicted between Type IIb and Pulselessness/Visual Problems, Type III and upper limb claudication/Pulselessness, Type IV and upper limb claudication/Hypertension, Type V with palpitation.

In terms of the ratio between positive and negative association with clinical Manifestations, regardless of statistical significance, certain tendencies can be observed regarding the clinical severity of each type. Precisely, Type I was associated through a positive estimate with 8 symptoms, meaning than an increase in Type I prevalence leads to a higher prevalence of these symptoms (Upper limb claudication, down limb claudication, fatigue, headache, pulselessness, visual problems, stroke, arthralgia), while with the remaining 5, there was a negative correlation (8/5). Type IIa had a positive estimate with 5 patient symptoms (upper limb claudication, down limb claudication, chest pain, stroke, vascular bruit) and a negative association with eight (5/8). Type IIb had a positive correlation with only one symptom (chest pain), while the rest of the manifestation were negatively connected (1/12). Type III had a positive estimate for 3 symptoms (chest pain, hypertension, vascular bruit), and a negative one for 10 (3/10). Furthermore, type IV presented with a positive association with 4 symptoms (chest pain, visual problems, hypertension, pulselessness), and with a negative estimate for 9 (4/9). Finally, 7 symptoms had a positive correlation with type V (fatigue, palpitation, headache, visual problems, hypertension, syncope, arthralgia), while only six had a negative one (7/6).

Therefore, after taking into consideration the entirety of the meta regression statistical data, regardless of statistical significance, Type I appears to be the most severe angiographic type of Takayasu arteritis, as its presence is associated with an increase in prevalence of 8 of the 13 assessed symptoms, followed by Type V. On the other hand, Type IIb can be characterized as the mildest from of Takayasu arteritis, as its presence is correlated with a decrease in prevalence for all but one patient symptom.

After taking into consideration only the statistically significant results from the meta regression, Type I and IIa do not have any significant association with clinical symptoms, Type IIb presents with 2 negative estimates, Type III with 2 negative, Type IV with one positive and one negative estimates, and Type V with one positive. Based on these results, an increased severity of type V can be roughly observed, while the more moderate clinical picture of type IIb is detectable. On the other hand, the severity of Type I is not observable at all this time around.

Thus, through a combined assessment of both statistically significant and non-significant results, the increased clinical severity of type V, as well as the modest clinical profile of type IIb and III can be deducted.

A retrospective cohort study by Bodakci et al. investigated the impact of Takayasu arteritis on pregnancy outcomes. The results and conclusions from this study partly agree with our findings, as they describe Type V as the most important risk factor for complications. More precisely, the presence of Type V had a greater association with hypertension, preeclampsia, eclampsia, and maturity compared to the rest of them [82]. Furthermore, a systematic review and meta-analysis by Misra et al. investigated the patient reported outcomes in Takayasu Arteritis. Among their results, they reported no statistically significant difference in fatigue in patients with Takayasu arteritis. This finding is in agreement with our results about this clinical symptom, as we also did not find any significant correlation between any angiographic types and patient fatigue [83]. Another systematic review and meta-analysis by Misra et explored, among others, the prevalence of Takayasu arteritis angiographic types as well as multiple clinical manifestations between pediatric-onset and adult-onset of Takayasu arteritis [84]. Their results showed that the pediatric-onset group, apart from having Type IV as the most common classification type, was associated with Hypertension and headache, in a significant way. On the other hand, Type I proved to be the most common type for the adult-onset group, while pulselessness and upper limb claudication were the most prominent associated clinical symptoms. Based on the aforementioned findings by Misra et al., our meta-analysis agrees on the presence of a positive association between Type IV and hypertension, whereas the rest of the results were not supported by statistically significant data.

To our knowledge, this systematic review with meta-analysis is the first to investigate and calculate the pooled prevalence percentage for each angiographic type of Takayasu arteritis, along with their variability across different areas and geographic modalities. Additionally, despite the existence of other reviews and meta-analyses regarding the clinical manifestations and risk factors for patients with Takayasu arteritis, this analysis is the first to explore the clinical severity of each angiographic type through the assessment of multiple different patient symptoms.

Thus, we believe that the classification of each affected patient into the six categories, determined by Hata, can provide clinical physicians with helpful information regarding the prediction of the clinical course of the patient, as well as the preparation for severe symptom management.

## Strengths

This analysis is strengthened by a clearly defined and independently executed study selection and data extraction process, that can reduce the risk of bias. Additionally, the use of a standardized and widely recognized classification system (Hata), rigorous statistical methodology including extensive pooled prevalences, sub-group analyses, univariate analyses, p-value corrections, comprehensive risk of bias and publication bias assessments (Hoy tool, LFK index, Peters’ test), and sensitivity analyses confirming the robustness of pooled estimates. Finally, the Benjamini–Hochberg procedure was applied in order to control the FDR of the p-values that were generated. The transparent use of validated tools and open-source statistical packages further enhances the reproducibility and reliability of the findings.

## Limitations

This systematic review and meta-analysis presented with a few limitations. Specifically, several studies were characterized as Moderate or High Risk according to the Hoy et al. tool for prevalence studies, while even though no study had major asymmetry, minor asymmetry was observed in some statistical results, implying a small study effect. Furthermore, High between-study heterogeneity persisted across most analyses, likely reflecting differences in imaging modalities, diagnostic criteria, and population characteristics (I^2^ > 70%). The geographic distribution of studies was also imbalanced, with a predominance of data from Asia and limited representation from other regions, which may affect the generalizability of results. There were some limitations to clinical data reporting, as not all studies reported clinical manifestations, resulting in a limited ability to explore the association between angiographic types and clinical patient symptoms.

## Conclusion

This systematic review and meta-analysis highlight the variable prevalence and clinical profiles associated with the different angiographic types of Takayasu arteritis, based on Hata’s classification. Type V emerged as the most common and clinically severe form, based on its positive associations with patient symptoms, which was also supported by statistically significant data. In contrast, Type IIb appeared to have the mildest clinical profile, with predominantly negative symptom correlations. While Type I demonstrated an apparent trend toward higher symptom burden, this was not supported by statistically significant findings. These results suggest that angiographic classification may offer meaningful insight into the clinical trajectory of Takayasu arteritis, potentially aiding in prognosis, early therapeutic planning, and anticipating the disease’s severity.

## Future research directions

Based on our results, we recommend more population studies, exploring the prevalence of angiographic types of Takayasu arteritis, as well as their clinical manifestations, in other geographical areas, which are typically not represented enough, such as Oceania. Additionally, further studies using certain diagnostic modalities, like conventional angiographies, should be performed so that there is more clinical data regarding the association between diagnostic method and angiographic types. Finally, studies with more detailed reporting of the clinical symptoms of all patients is crucial, since it creates more opportunities to investigate and research further characteristics of a patient’s clinical trajectory.

## Supplementary Information

Below is the link to the electronic supplementary material.

Supplementary Material 1

Supplementary Material 2

Supplementary Material 3

## Data Availability

No datasets were created.
