# Supplementary material for: Prevalence and clinical severity of takayasu arteritis angiographic types: a systematic review with meta-analysis
Source: Rheumatol Int. 2025 Sep 22;45(10):231. doi: 10.1007/s00296-025-05983-4 (PMC12454456; doi:10.1007/s00296-025-05983-4)
Supplement: Supplementary file 2 — Supplementary Material 2 [file 296_2025_5983_MOESM2_ESM.docx]

Table 1: Risk of Bias Assessment of the included studies based on Hoy et al. tool

| Study Name | Representation of the target population | Sampling Frame | Random Selection or Census | Non-Response Bias |
| --- | --- | --- | --- | --- |
| Gudbrandson | Low Risk | Low Risk | Low Risk | Low Risk |
| Gloor | Moderate Risk | Moderate Risk | Low Risk | Moderate Risk |
| Saritas | Moderate Risk | Moderate Risk | Low Risk | Low Risk |
| Dreyer | Low Risk | Low Risk | Low Risk | Low Risk |
| Soto | Moderate Risk | Moderate Risk | High Risk | Low Risk |
| Karageorgaki | Moderate Risk | Low Risk | Low Risk | Low Risk |
| Bicakcigil | Low Risk | Low Risk | Low Risk | Low Risk |
| Schmidt | Moderate Risk | Low Risk | Low Risk | Low Risk |
| Lim | Moderate Risk | Low Risk | Low Risk | Low Risk |
| Sato | Moderate Risk | Low Risk | Low Risk | Low Risk |
| Zhou | Moderate Risk | Low Risk | Low Risk | Low Risk |
| Sheikzadeh | Moderate Risk | Moderate Risk | Low Risk | Moderate Risk |
| Canas | High Risk | High Risk | High Risk | High Risk |
| Setty | Moderate Risk | Moderate Risk | Low Risk | Low Risk |
| Sun | Moderate Risk | Moderate Risk | Low Risk | Low Risk |
| Lee | Low Risk | Low Risk | Low Risk | Low Risk |
| Cong | High Risk | High Risk | Moderate Risk | Low Risk |
| Park | High Risk | High Risk | Moderate Risk | Low Risk |
| Suwanela | Moderate Risk | High Risk | Moderate Risk | Low Risk |
| Alvarez | Moderate Risk | Moderate risk | Low Risk | Low Risk |
| Ong | Low Risk | Low Risk | Low Risk | Low Risk |
| Watanabe | Low Risk | Low Risk | Low Risk | Moderate Risk |
| Esen | Moderate Risk | Moderate Risk | Low Risk | Low Risk |
| Markin | Moderate risk | Low Risk | Low Risk | Low Risk |
| Comarmond | Moderate risk | Moderate Risk | Low Risk | Moderate Risk |
| Danda | Moderate Risk | Moderate Risk | Low Risk | Low Risk |
| Kong | Moderate risk | Low Risk | Low Risk | Low Risk |
| Karabacak | Moderate risk | Low Risk | Low Risk | Low Risk |
| Oliveira | Moderate Risk | Moderate Risk | High risk | Moderate Risk |
| Mahdavi | Moderate Risk | Moderate Risk | Low Risk | Moderate Risk |
| Petrovic | High Risk | Moderate risk | Low Risk | Low Risk |
| Arnaud | Moderate risk | Low Risk | Low Risk | Low Risk |
| Tamartash | Moderate Risk | Moderate Risk | Low Risk | Low Risk |
| Li | High Risk | High Risk | High Risk | Low Risk |
| Goel | Moderate Risk | High risk | Moderate Risk | Low Risk |
| Kim | Moderate Risk | Moderate Risk | Low Risk | Low Risk |
| De Paula | High Risk | High Risk | Low Risk | Low Risk |
| Eleftheriou | High Risk | High Risk | Low Risk | Low Risk |
| Li J | High Risk | High Risk | Low Risk | Low Risk |
| Cheng | Moderate Risk | Moderate risk | Low Risk | Low Risk |
| Figueiroa | Moderate risk | Moderate Risk | High Risk | Low Risk |
| Zhang | Moderate Risk | Moderate risk | Low Risk | Low Risk |
| Wong | Moderate Risk | Low Risk | Low Risk | Low Risk |
| Zhang Y | Moderate risk | Moderate Risk | Low Risk | Low Risk |
| Khan | Moderate risk | Moderate Risk | Low Risk | Low Risk |
| Clemente | Moderate risk | Moderate Risk | Low Risk | Low Risk |
| Johnson | High Risk | Moderate Risk | Low Risk | Low Risk |
| Lei | High Risk | Moderate Risk | Low Risk | Low Risk |
| Kelesoglu | High Risk | Moderate Risk | Low Risk | High Risk |
| Hegde | High Risk | High risk | Low Risk | Moderate Risk |
| Xi | High Risk | Moderate Risk | Low Risk | Low Risk |
| Misra | High Risk | Moderate Risk | Low Risk | Low Risk |
| Zhang | High Risk | Moderate Risk | Low Risk | Low Risk |
| Misra | High risk | Moderate Risk | Low Risk | Low Risk |
| Mukoyoma | High Risk | Moderate Low Risk | Low Risk | Low Risk |
| Zhang | High Risk | High Risk | Low Risk | Low Risk |
| He | High Risk | High Risk | High Risk | Moderate Risk |
| Ren | Moderate Risk | Moderate Risk | High Risk | Low Risk |
| Li | Moderate Risk | Moderate Risk | Low Risk | Low Risk |
| Kwon | Moderate Risk | Low Risk | Low Risk | Low Risk |
| Ma | Moderate Risk | Low Risk | Low Risk | Low Risk |
| Kalfa | Moderate Risk | Low Risk | Moderate Risk | Low Risk |
| Fan | Moderate Risk | Moderate Risk | Low Risk | Low Risk |
| Chen | Moderate Risk | Moderate Risk | Low Risk | Low Risk |
| Wang | High Risk | High Risk | Low Risk | Low Risk |
| Kong | High Risk | High Risk | High Risk | Moderate Risk |

Table 2: Risk of Bias Assessment of the included studies based on Hoy et al. tool (continuation)

| Study Name | Data collection directly from subjects | Acceptable case definition | Reliable and valid measurement instrument | Same mode of data collection for all subjects | Appropriate length of the shortest prevalence period | Numerator and denominator appropriate |
| --- | --- | --- | --- | --- | --- | --- |
| Gudbrandson | Low Risk | Low Risk | Low Risk | Low Risk | Low Risk | Low Risk |
| Gloor | Low Risk | Low Risk | Low Risk | Moderate Risk | Low Risk | Moderate Risk |
| Saritas | Moderate Risk | Low Risk | Moderate Risk | Low Risk | Low Risk | Low Risk |
| Dreyer | Moderate Risk | Low Risk | Low Risk | Low Risk | Low Risk | Low Risk |
| Soto | Moderate risk | Low Risk | Low Risk | Low Risk | High Risk | Moderate Risk |
| Karageorgaki | Moderate Risk | Low Risk | Low Risk | Low Risk | Low Risk | Moderate Risk |
| Bicakcigil | Low Risk | Low Risk | Low Risk | Low Risk | Low Risk | Low Risk |
| Schmidt | Moderate Risk | Low Risk | Low Risk | Low Risk | Low Risk | Low Risk |
| Lim | Moderate Risk | Low Risk | Low Risk | Low Risk | Low Risk | Low Risk |
| Sato | Moderate Risk | Low Risk | Moderate Risk | High Risk | High Risk | Low Risk |
| Zhou | Low Risk | Low Risk | Low Risk | Low Risk | Low Risk | Low Risk |
| Sheikzadeh | Low Risk | Low Risk | Low Risk | Low Risk | Low Risk | Moderate Risk |
| Canas | High Risk | Low risk | High Risk | Low Risk | Low Risk | Low Risk |
| Setty | Low Risk | Low Risk | Low Risk | Low Risk | Moderate Risk | Low Risk |
| Sun | High Risk | Low Risk | Low Risk | Moderate Risk | High Risk | Low Risk |
| Lee | Low Risk | Low Risk | Low Risk | Low Risk | Low Risk | Low Risk |
| Cong | High Risk | Low Risk | Low Risk | Moderate Risk | High Risk | Low Risk |
| Park | High Risk | Low Risk | Low Risk | Moderate Risk | N/A | Low Risk |
| Suwanela | Moderate Risk | Moderate Risk | Low Risk | Moderate risk | Low Risk | Low Risk |
| Alvarez | Moderate Risk | Low Risk | Low Risk | Low Risk | Low Risk | Low Risk |
| Ong | Moderate Risk | Low Risk | Low Risk | Low Risk | Low Risk | Low Risk |
| Watanabe | Moderate Risk | Low Risk | Moderate Risk | Low Risk | Low Risk | Moderate Risk |
| Esen | Low Risk | Low Risk | Moderate Risk | Low Risk | Low Risk | Low Risk |
| Markin | Moderate Risk | Low Risk | Moderate Risk | Low Risk | Low Risk | Low Risk |
| Comarmond | Moderate Risk | Low Risk | Low Risk | Low Risk | Low Risk | Low Risk |
| Danda | Moderate Risk | Low Risk | Low Risk | Low Risk | Low Risk | Low Risk |
| Kong | Moderate Risk | Low Risk | Low Risk | Low Risk | Low Risk | Low Risk |
| Karabacak | Moderate Risk | Low Risk | Low Risk | Low Risk | Low Risk | Low Risk |
| Oliveira | Low Risk | Low Risk | Low Risk | Low Risk | Low Risk | Low Risk |
| Mahdavi | Low Risk | Low Risk | Low Risk | Low Risk | Low Risk | Low Risk |
| Petrovic | Low Risk | Low Risk | Low Risk | Low Risk | Low Risk | Low Risk |
| Arnaud | Low Risk | Low Risk | Low Risk | Low Risk | Low Risk | Low Risk |
| Tamartash | Low Risk | Low Risk | Low Risk | Moderate risk | Low Risk | Low Risk |
| Li | Low Risk | Low Risk | Low Risk | Low Risk | N/A | Low Risk |
| Goel | Low Risk | Low Risk | Low Risk | Moderate risk | Low Risk | Low Risk |
| Kim | Low Risk | Low Risk | Low Risk | Low Risk | Low Risk | Low Risk |
| De Paula | Low Risk | Low Risk | Low Risk | Low Risk | Low Risk | Low Risk |
| Eleftheriou | Low Risk | Low Risk | Low Risk | Low Risk | Low Risk | Low Risk |
| Li J | Moderate Risk | Low Risk | Low Risk | Low Risk | Low Risk | Low Risk |
| Cheng | Low Risk | Low Risk | Low Risk | Low Risk | Low Risk | Low Risk |
| Figueiroa | Low Risk | Low Risk | Low Risk | Low Risk | Low Risk | Low Risk |
| Zhang | Low Risk | Low Risk | Low Risk | Low Risk | Low Risk | Low Risk |
| Wong | Moderate Risk | Low Risk | Low Risk | Moderate Risk | Low Risk | Low Risk |
| Zhang Y | Moderate Risk | Low Risk | Low Risk | Low Risk | Low Risk | Low Risk |
| Khan | Low Risk | Low Risk | Low Risk | Low Risk | Low Risk | Low Risk |
| Clemente | Low Risk | Low Risk | Moderate Risk | Moderate Risk | Low Risk | Low Risk |
| Johnson | Moderate Risk | Low Risk | Moderate Risk | Low Risk | Low Risk | Low Risk |
| Lei | Moderate Risk | Low Risk | Moderate Risk | Low Risk | Low Risk | Low Risk |
| Kelesoglu | Low Risk | Low Risk | Low Risk | Low Risk | Low Risk | Low Risk |
| Hegde | Moderate Risk | Low Risk | Low Risk | Low Risk | Low Risk | Low Risk |
| Xi | Low Risk | Low Risk | Low Risk | Low Risk | Low Risk | Low Risk |
| Misra | Low Risk | Low Risk | Low Risk | Low Risk | Low Risk | Low Risk |
| Zhang | Low Risk | Low Risk | Low Risk | Low Risk | Low Risk | Low Risk |
| Misra | High Risk | Low Risk | Low Risk | Low Risk | Low Risk | Low Risk |
| Mukoyoma | High risk | Low Risk | Low Risk | Moderate Risk | Low Risk | Low Risk |
| Zhang | High Risk | Low Risk | Low Risk | Moderate Risk | Moderate Risk | Low Risk |
| He | Low Risk | Low Risk | Low Risk | Low Risk | Low Risk | Low Risk |
| Ren | Low Risk | Low Risk | Low Risk | Low Risk | N/A | Low Risk |
| Li | Moderate Risk | Low Risk | Low Risk | Low Risk | Low Risk | Low Risk |
| Kwon | Low Risk | Low Risk | Low Risk | Low Risk | Low Risk | Low Risk |
| Ma | Low Risk | Low Risk | Low Risk | Low Risk | Low Risk | Low Risk |
| Kalfa | Moderate Risk | Low Risk | Low Risk | Low Risk | N/A | Low Risk |
| Fan | Low Risk | Low Risk | Low Risk | Moderate Risk | Low Risk | Low Risk |
| Chen | Low Risk | Low Risk | Low Risk | Moderate risk | Low Risk | Low Risk |
| Wang | High Risk | Low Risk | Low Risk | Low Risk | Low Risk | Low Risk |
| Kong | Low Risk | Low Risk | Low Risk | Low Risk | Moderate Risk | Low Risk |
