# Supplementary material for: Prevalence and clinical severity of takayasu arteritis angiographic types: a systematic review with meta-analysis
Source: Rheumatol Int. 2025 Sep 22;45(10):231. doi: 10.1007/s00296-025-05983-4 (PMC12454456; doi:10.1007/s00296-025-05983-4)

Type I

Linear regression test of funnel plot asymmetry

Test result: t = 1.27, p-value = 0.2103

Bias estimate: 7.8891 (SE = 6.2337)


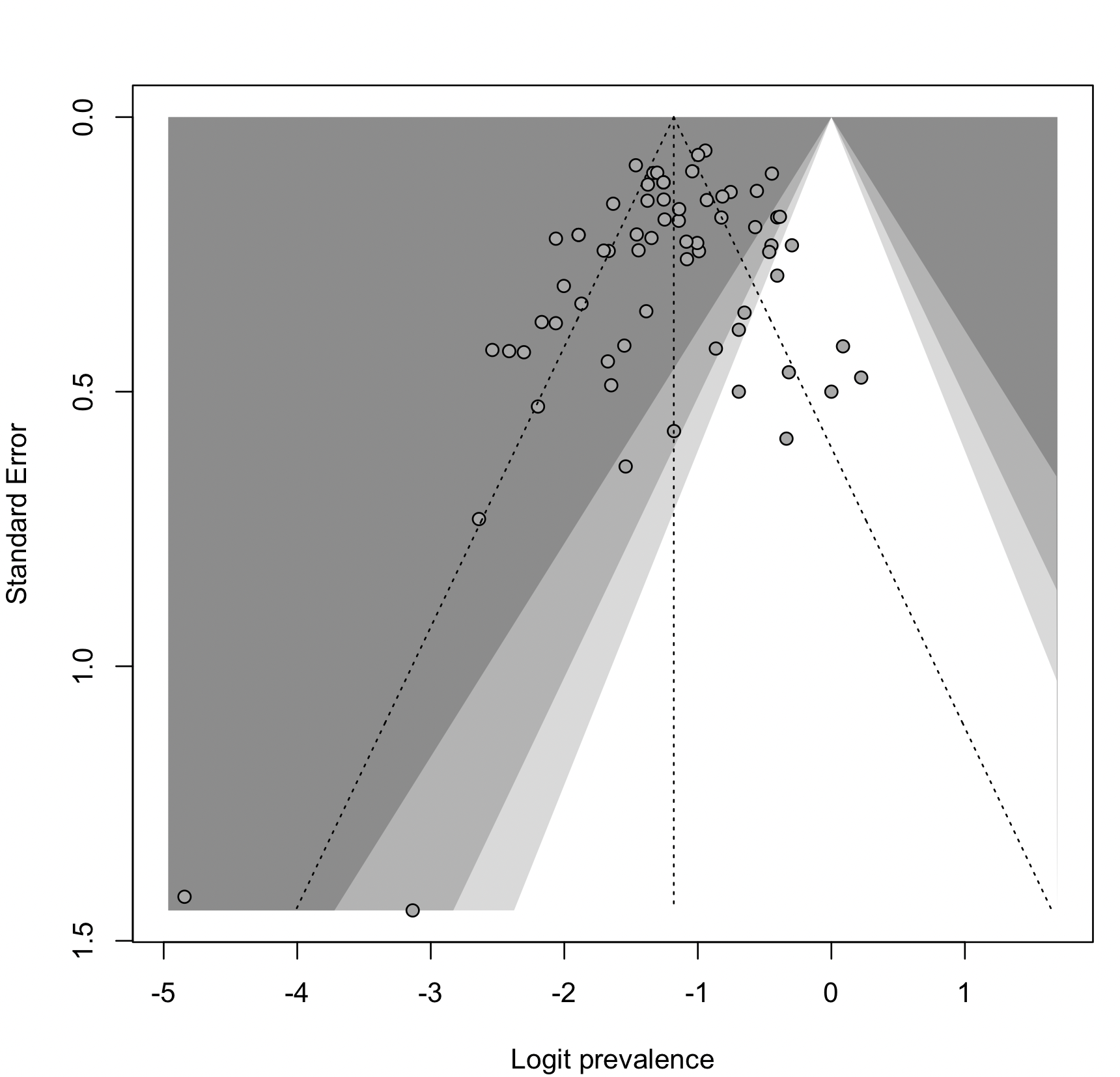


Type IIa

Linear regression test of funnel plot asymmetry

Test result: t = 0.70, p-value = 0.4876

Bias estimate: 6.1368 (SE = 8.7855)


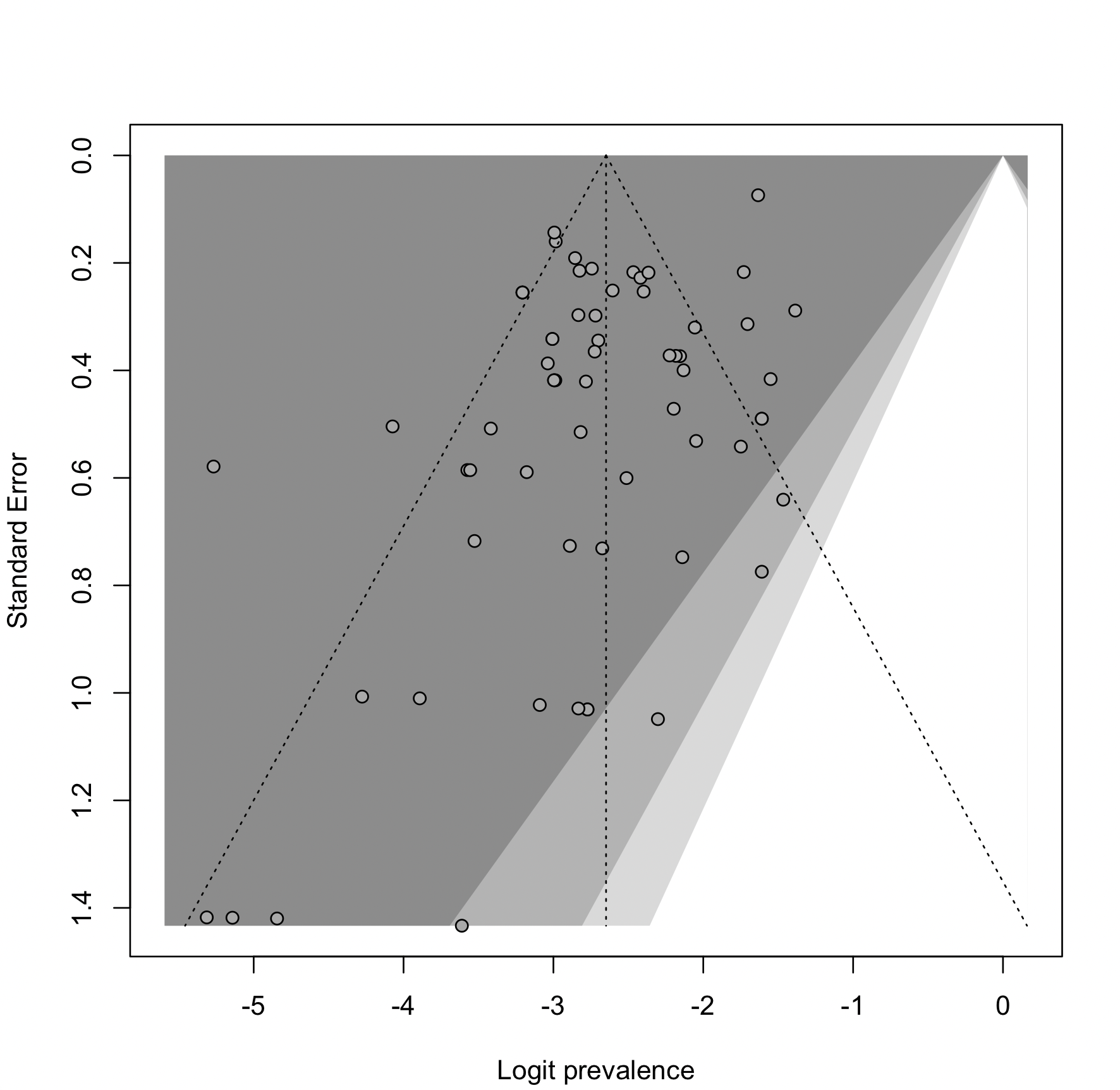


Type IIb

Linear regression test of funnel plot asymmetry

Test result: t = 1.00, p-value = 0.3228

Bias estimate: 9.8003 (SE = 9.8256)


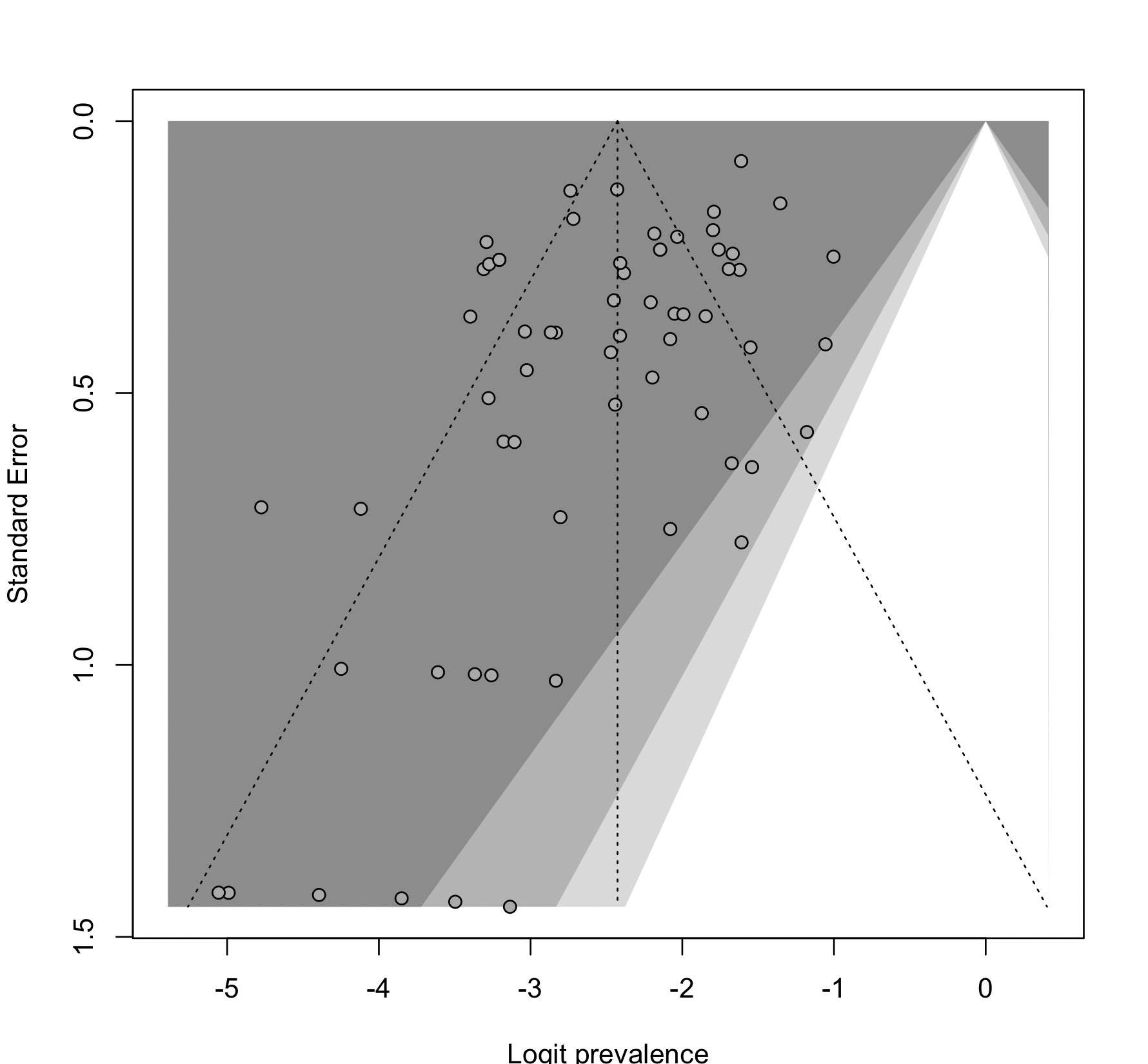


Type III

Linear regression test of funnel plot asymmetry

Test result: t = 1.64, p-value = 0.1077

Bias estimate: 16.8797 (SE = 10.3186)


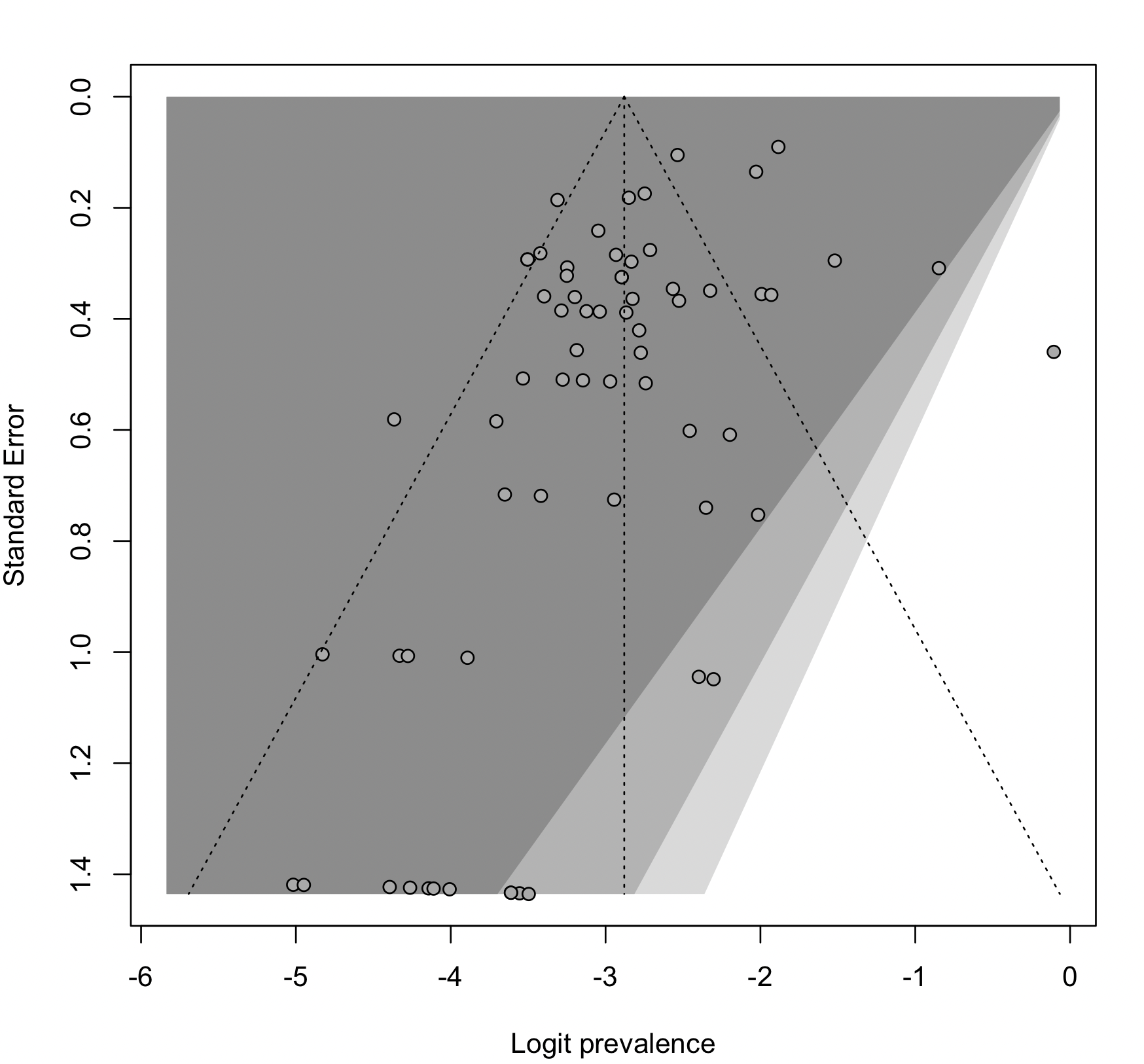


Type IV

Linear regression test of funnel plot asymmetry

Test result: t = 1.58, p-value = 0.1184

Bias estimate: 17.7018 (SE = 11.1750)


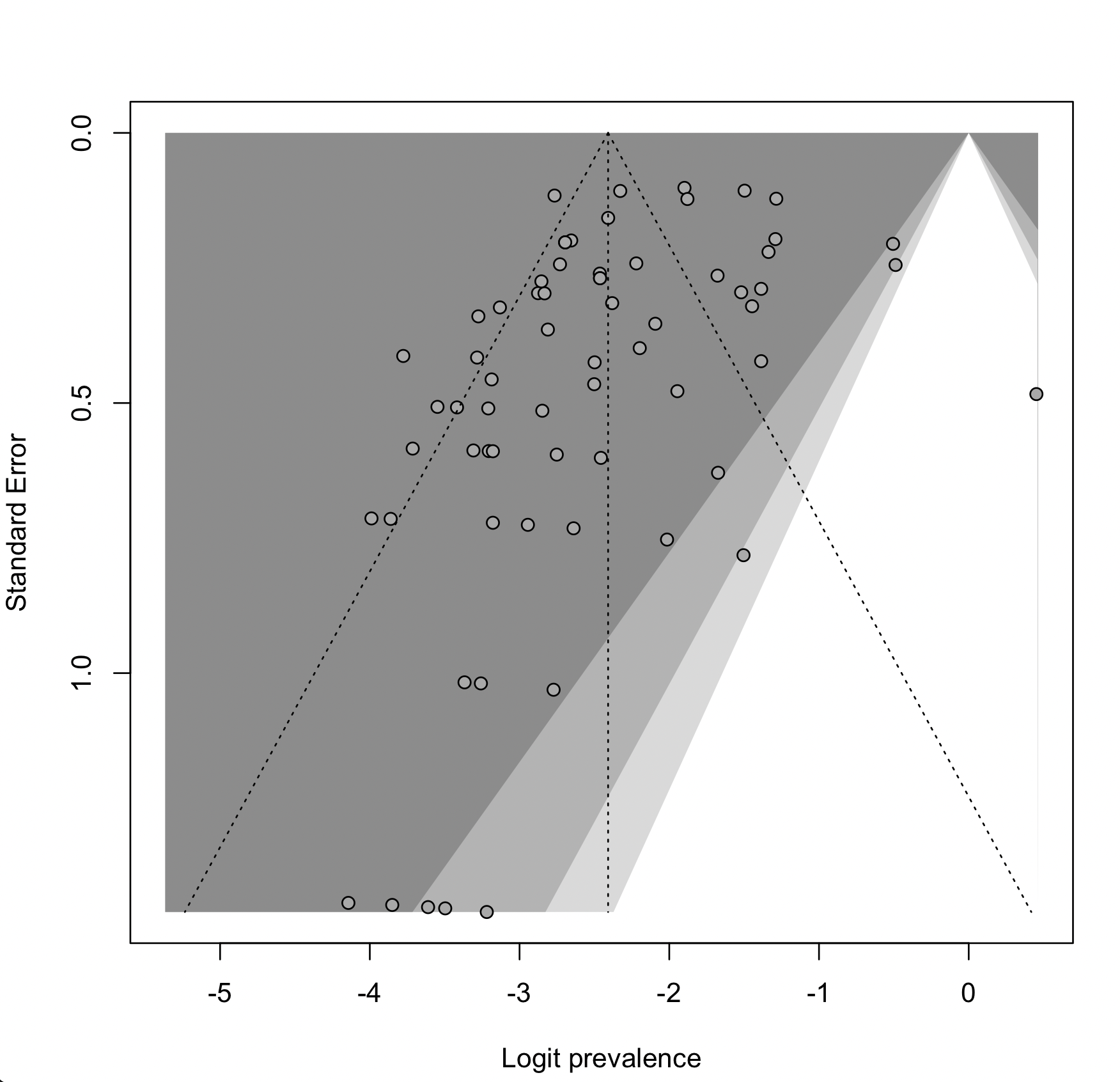


Type V

Linear regression test of funnel plot asymmetry

Test result: t = -0.28, p-value = 0.7790

Bias estimate: -2.2969 (SE = 8.1531)


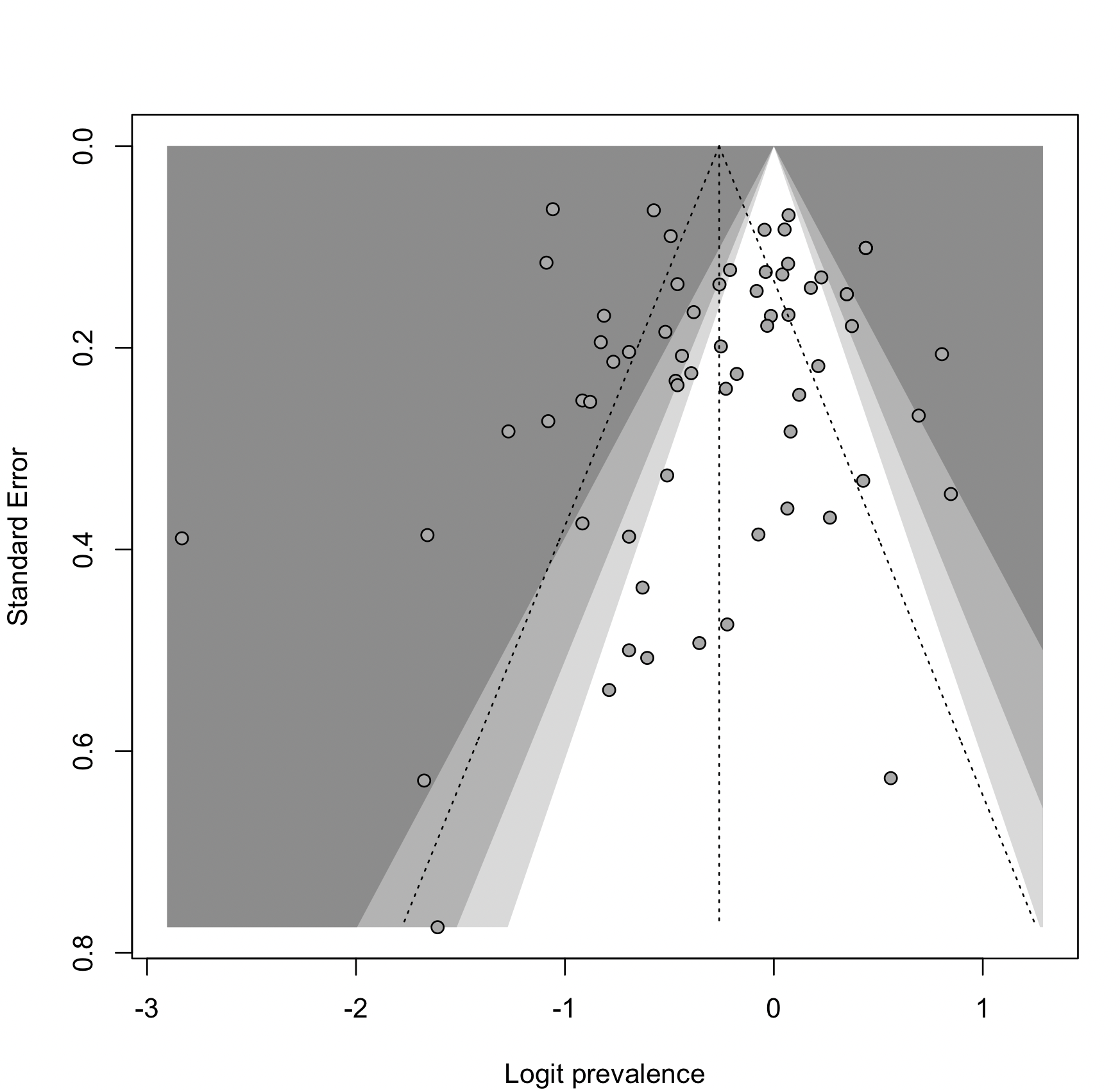

Supplement: Supplementary file 3 — Supplementary Material 3 [file 296_2025_5983_MOESM3_ESM.docx]
